# Discovering geothermal supercritical fluids: a new frontier for seismic exploration

**DOI:** 10.1038/s41598-017-15118-w

**Published:** 2017-11-06

**Authors:** Nicola Piana Agostinetti, Andrea Licciardi, Davide Piccinini, Francesco Mazzarini, Giovanni Musumeci, Gilberto Saccorotti, Claudio Chiarabba

**Affiliations:** 1 Department of Geodynamics and Sedimentology, University of Vienna, Vienna, Austria; 2 Geophysics Section, School of Cosmic Physics, Dublin Institute for Advanced Studies, Dublin, Ireland; 3 Istituto Nazionale di Geofisica e Vulcanologia, Centro Nazionale Terremoti, Rome, Italy; 4 Géosciences Rennes, Univerisity of Rennes 1, Rennes, France; 5 Istituto Nazionale di Geofisica e Vulcanologia, Sezione di Pisa, Pisa, Italy; 6 Dipartimento di Scienze della Terra, Universita’ di Pisa, Pisa, Italy

## Abstract

Exploiting supercritical geothermal resources represents a frontier for the next generation of geothermal electrical power plant, as the heat capacity of supercritical fluids (SCF),which directly impacts on energy production, is much higher than that of fluids at subcritical conditions. Reconnaissance and location of intensively permeable and productive horizons at depth is the present limit for the development of SCF geothermal plants. We use, for the first time, teleseismic converted waves (i.e. receiver function) for discovering those horizons in the crust. Thanks to the capability of receiver function to map buried anisotropic materials, the SCF-bearing horizon is seen as the 4km-depth abrupt termination of a shallow, thick, ultra-high (>30%) anisotropic rock volume, in the center of the Larderello geothermal field. The SCF-bearing horizon develops within the granites of the geothermal field, bounding at depth the vapor-filled heavily-fractured rock matrix that hosts the shallow steam-dominated geothermal reservoirs. The sharp termination at depth of the anisotropic behavior of granites, coinciding with a 2 km-thick stripe of seismicity and diffuse fracturing, points out the sudden change in compressibility of the fluid filling the fractures and is a key-evidence of deep fluids that locally traversed the supercritical conditions. The presence of SCF and fracture permeability in nominally ductile granitic rocks open new scenarios for the understanding of magmatic systems and for geothermal exploitation.

## Introduction

Exploiting supercritical geothermal resources represents a frontier for the next generation of geothermal electrical power plant^1–3^, as the heat capacity of SCF is much higher than that of fluids at subcritical conditions^4,5^. Reconnaissance and location of SCF horizons at depth, where fluids locally traverse the supercritical conditions^6^, is the present limit for the development of SCF geothermal plants. Moreover, SCF resources needs to be associated to significant fracture permeability in nominally ductile granitic rocks^7^ for representing potential productive horizons. Since the last century, shallow crust (0–1 km) high-entalphy (>150 °C) geothermal reservoir is one of the oldest exploited renewable sources of energy^8^. Steam entrapped within the rock matrix is used for electricity production and district heating. In the last decade, the exploitation of fluids close to the supercritical conditions (i.e. 374 °C and 22.1 MPa, for H_2_O) has increased, because it can lead to a ten-fold increase in energy extraction^1^. The Icelandic project IDDP-1 encountered rhyolitic magma at shallow depth (>900 °C at 2104 m depth) and achieved flow-rates for a SCF of up to 50 kg/s^2^. This reinforced the interest in SCF geothermal resources and stimulated a number of studies for solving fundamental issues related to the exploitation of SCF. Pilot-projects have been developed on top of well-known SCF reservoirs^9^ for testing engineering and drilling solutions. Less attention has been paid to exploration of new SCF resources. The exploration phase is considered the most important aspect for reducing the risk in the development of a geothermal plant^10–12^ but, nevertheless, no SCF reservoir has been successfully explored until today and there is little constraint on the seismic signature of SCF within shallow-crustal magmatic intrusions, limiting the potential for new discoveries.

**Figure 1:**
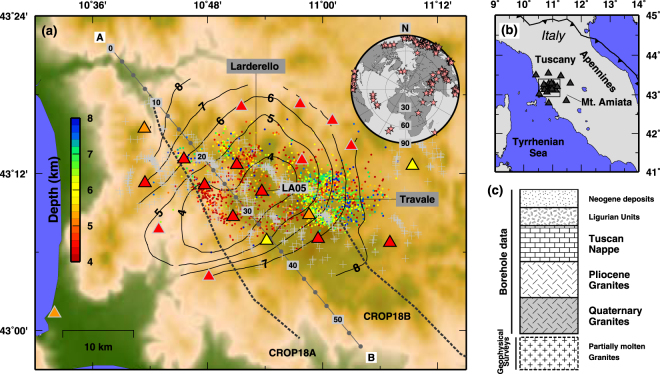
Map of the study area. (**a**) Seismic stations deployed across the Larderello geothermal field (triangles): GAPSS experiment -red; a NSF-funded project, RETREAT - orange; permanent Italian Seismic Network, ISN - yellow. Seismic stations used for computing the profiles in Fig. 2 are draw with a black outline. Grey crosses indicate piercing-points of teleseismic rays at 15 km depth. The grey line displays the trace of the profile used in Figs 2 and 3, numbers indicates km along the profile. Traces of the CROP18A and B active seismic profiles are also shown. Contour lines indicate depth of the k-horizon in the area, from active seismics^13^. Coloured dots show focal depth of seismic events located using GAPSS data. Inset: back-azimuthal distribution of the teleseisms selected for station LA05. (**b**) Complete map of the seismic stations used in this study. A black box indicates the area shown in (**a**). Grey triangles indicate seismic stations. (**c**) Simplified lithostratigraphy for the Larderello area^18^. Figures have been created using GMT^51^.

The Lardello geothermal field, the oldest worldwide^13^, is the optimal place for experimenting techniques in SCF exploration (Fig. 1). Larderello is a so called *steam-dominated* geothermal field, where the shallow-crustal rocks host a system of vapor-filled fractures, mapped at the surface^14^. The presence of SCF in the Larderello field has been suggested after the un-successful drilling of the S. Pompeo well, where extrapolated bottom temperature and pressure reached >400 °C and 24.0 MPa^9,15,16^. Numerical modeling of the Larderello reservoirs also showed plausible P-T conditions for the presence of SCF, for a wide range of saline brines (NaCl concentration as high as 25%)^17^. Many authors suggested that SCF-bearing horizons in Larderello coincide with the energetic signals, i.e. bright-spots, found throughout the geothermal field by active seismic surveys (collectively called “k-horizon”, Fig. 1a)^18^. The k-horizon has been mapped all across the area and shows a clear upraise where the heat flow is higher (increasing from 200 to ~1000 mW/m^2^) and the SCF conditions can be reached at shallow depths^16,18^.

The transition from sub-critical vapor to super-critical fluid conditions comes together with a sharp decrease in fluid compressibility^6^, resulting in an abrupt change in the seismic properties of the rock volume hosting the SCF. In fact, rocks hosting vapor-filled fractures behaves differently from rocks hosting fluid-filled fractures, during the propagation of seismic P-waves^19^. In the first case vapor-filled fractures give rise to a extremely pronounced P-wave anisotropy in the rock volume, in the latter case the rocks almost behave as an isotropic medium. Thus, the sharp termination at depth of a highly anisotropic, vapor-filled rock volume can mark the transition to SCF conditions. We exploit this characteristic for mapping the SCF-bearing horizons, using recordings of teleseismic converted waves, i.e. receiver function, RF^20^. RF analysis has been widely used to map anisotropic materials at depth^21,22^. Sophisticated tools have been developed for extracting from RF data-sets the signals related to anisotropy^23,24^. In this study, we image the SCF-bearing horizons as the sharp termination of highly anisotropic materials at depth, found by means of RF analysis and modeling. The position of SCF-bearing horizons at depth is also compared with precisely located miscroseismicity to evaluate the presence of significant fracture permeability.

## Results

The knowledge of SCF physical properties and of the geometry of SCF-bearing horizons is straightforward to enhance their geothermal exploitation. Results from the analysis of teleseismic data recorded from broadband stations installed across the Larderello field furnish a clear seismic evidence of SCF-bearing horizons in a geothermal area. Here we briefly illustrate the seismic data and the methodology used to map the SCF-bearing horizons, leaving the details to the Method section. The transition from sub-critical vapor to super-critical fluid concides with an approximately two order of magnitude decrease in fluid compressibility (or, conversely, a two order of magnitude increase in fluid bulk modulus *κ*_*f*_). This variation in compressibility strongly affects P-wave seismic anisotropy related to the rocks hosting the vapor-filled fractures^19^, and, thus, the transition to SCF can be spotted out by means of the analysis of seismic data. Teleseismic waveforms are routinely used to compute RF data-sets, i.e. series of teleseismic P-to-s converted waves generated at near-receiver seismic discontinuities^20^. A RF data-set contains information about the presence of anisotropy at depth in terms of 2*π* periodic amplitude variations of such P-to-s converted waves, as a function of the direction of the incoming P-wave^25^. Extracting periodic signals, so called *angular harmonics*, from RF data-set gives the opportunity to map the boundary of anisotropic materials at depth^23^.

Here we analyse teleseismic waveforms acquired during the Geothermal Area Passive Seismic Sources, GAPSS, seismic experiment^26^, complemented with data from temporary and permanent seismic networks. We analyse RF data-sets for retrieving energetic arrivals on the second k = 1 angular harmonics, not matched by similar phases on the first k = 0 angular harmonics, which represent a clear evidence of a change in anisotropic behavior of the rocks at depth^24^. We also make use of RF modeling to measure the strength of anisotropy of the rock volume^27^. Given the sharp change in fluid compressibility for fluid traversing the SCF conditions^6^, we related the change in the anisotropic behavior of the rocks to the transition of the fluid filling the fractures in the rock matrix, from sub-critical vapor to super-critical fluid state.

**Figure 2:**
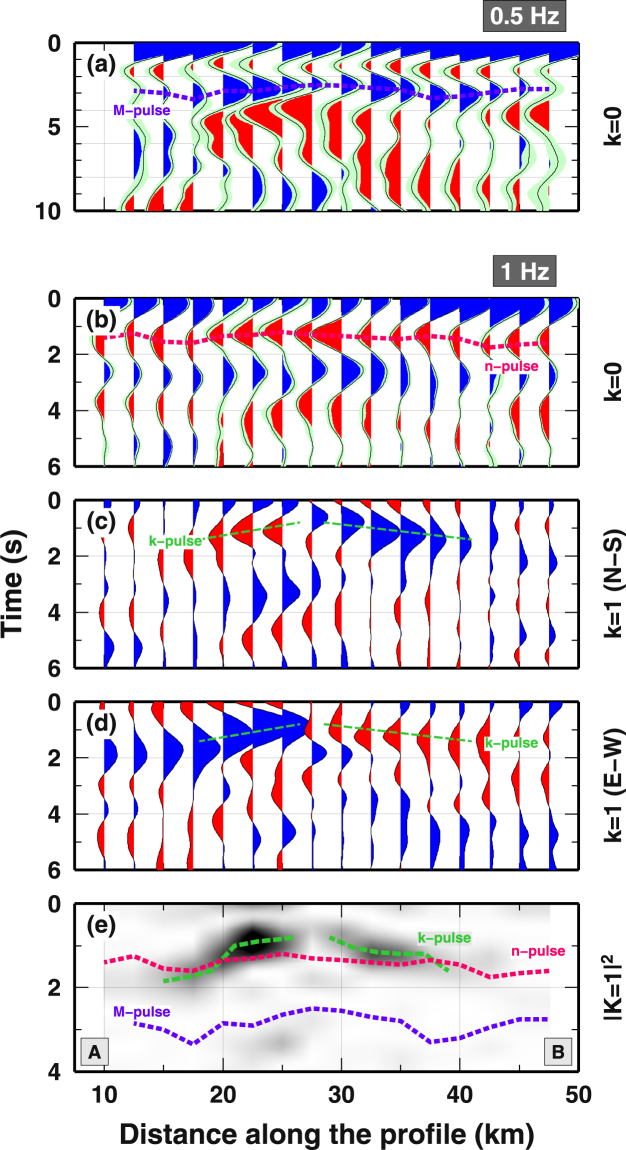
CCP image of the RF data-sets projected along the profile AB in Fig. 1. (**a**) k = 0 harmonics of the RF data-set at 0.5 Hz frequency. A dashed blue line indicates the time-position of the main positive Ps phase (M-pulse). (**b**) k = 0 harmonics of the RF data-set at 1 Hz. A dashed red line indicates the main negative Ps phase (n-pulse). (**c**) k = 1 harmonics of the RF data-set at 1 Hz, North-South component. (**d**) k = 1 harmonics of the RF data-set at 1 Hz, East-West component. In panels (c) and (d), a green dashed line show main pulses with positive and negative amplitude for k = 1 harmonics (k-pulse). (**e**) Energy on the k = 1 harmonics at 1 Hz as the sum of the squared N-S and E-W components. Blue and red dashed lines from panels (a) and (b). Green dashed lines indicate maximum of the energy (k-pulse). Figures have been created using GMT^51^.

Teleseismic waves have been used to reconstruct a Common-Conversion Point (CCP) image of the subsurface with RF analysis. The CCP image has been decomposed in its angular harmonics to separate the isotropic and the anisotropic contributions^22^ (see Methods, and Supplementary Figures S1 and S2) and presented along a NW-SE profile (Fig. 2). RF data have been also interpolated over the entire area to show the axis-symmetric characteristic of the main arrivals (Supplementary Figures S3, M1 and M2). The first harmonics, k = 0, is sensitive to the bulk change in shear-wave velocity (*V*_*s*_) at depth. At low frequency (0.5 Hz) the signal is dominated by the P-to-s converted waves from the Mohorovic (Moho) discontinuity, seen as a positive arrival between 2.8 and 3.3 s, up-doming in the center of the Larderello area to 2.5 s (marked as “M-pulse” in Fig. 2a). At higher frequency (1.0 Hz), the k = 0 harmonics along the profile displays a clear negative arrival between 1.3 and 1.7 s, suggesting a significant velocity reduction at depth, with a similar dome-like geometry following the M-pulse seen at low frequency (marked as “n-pulse” in Fig. 2b).

The k = 1 harmonics, a proxy for the presence of anisotropic materials^28^, shows a single pulse at 0.8–1.8 s which flips its polarity at 25–30 km along the profile, on both normal components (marked as k-pulse in Fig. 2c,d). The k-pulse displays the same dome-like pattern as for the M-pulse. Notably, the k-pulse arrives 0.4 s earlier between 20 and 30 km along the profile, with respect to the n-pulse, indicating that two separated seismic interfaces are responsible for the k- and n- pulses in the center of the dome. The presence of high amplitude arrival at 0 s suggest pervasive anisotropy in the shallow crust. Summarizing, in the center of the dome arrivals related to anisotropic materials (on k = 1 harmonics) are decoupled from arrivals related to bulk seismic velocity jumps (on k = 0 harmonics). The main arrivals at higher frequency display a 15 km-wide dome-like structure which follows the geometry of the Moho up-doming seen at lower frequency.

**Figure 3:**
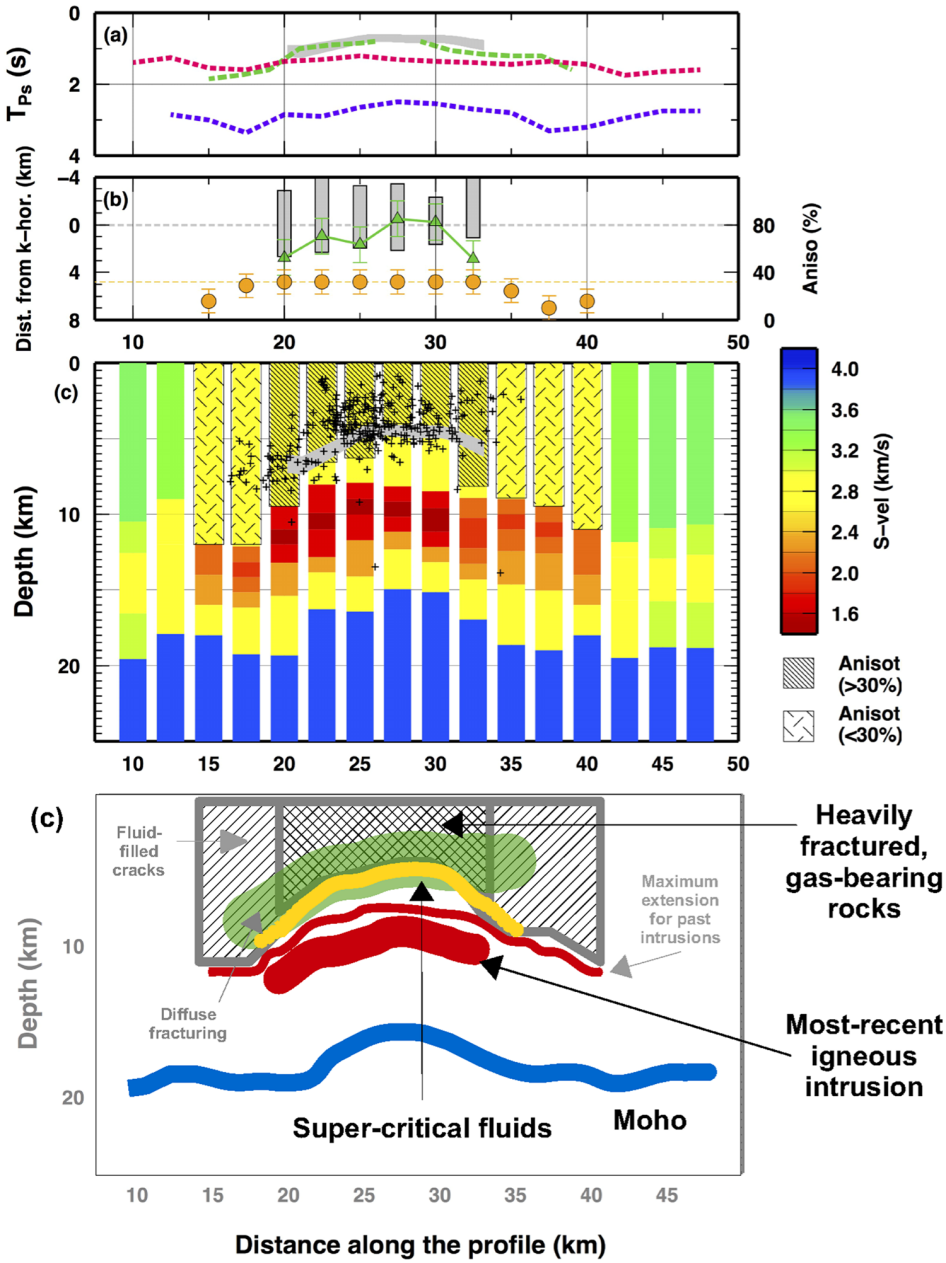
(**a**) Comparison of the M-, n- and k- pulses with the “k-horizon”. For a precise comparison, we compute the time-delay between the P wave and S converted wave (Tps) of the “k-horizon” from the Two-Way-Time (TWT) data (see methodological details in Supplementary Figure S4). A grey band indicate the minimum and maximum Tps of the k-horizon along the profile as computed using two end-member models. Dashed lines as in Fig. 2e. (**b**) Depth-distribution of seismicity along the profile, reported as distance from the k-horizon. Grey bars show the +/−2std intervals for the depth of the seismic events relative to the k-horizon. Green triangles and the green dashed line show the difference in depth between the k-horizon and the bottom of the ultra-high anisotropic volume along the profile. Anisotropic values along the profiles are shown as orange circles. An orange dashed line shows the 32% anisotropic level given by vapor-bearing microcracks^19^. (**c**) S-wave velocity model for each point along the profile AB in Fig. 1. Colors indicate S-wave velocity. Texture indicates the area where highly anisotropic materials are present. A grey area delineated the minimum and maximum values for the k-horizon at depth, as reported in Supplementary Figure S4f. Black crosses report the depth of the seismic events occurred along the profile. (**d**) Schematic interpretation of our observations and modeling for the Larderello geothermal field. Figures have been created using GMT^51^.

The shallow seismic discontinuity (k-pulses) coincides with the “k-horizon”, energetic arrivals identified by active seismic surveys^16^, assumed to be generated by over-pressured fluids heated to near supercritical conditions^18^. The two-way time (TWT) of the “k-horizon” varies between 1.4 and 3.6 s. We mapped the TWT iso-contours of the k-horizon as seen by 396 active seismic surveys^29^ and migrated the TWT along our profile using two end-member models (Supplementary Figure S4). The strong correlation between the “k-horizon” and the k-pulse along the profile (Fig. 3a) indicates a link between the anisotropic signature in the RF data and the bright spots in active seismic surveys.

A Monte Carlo inversion of the RF is performed to quantify the seismic properties of the Larderello geothermal field (Fig. 2b,c and Supplementary Figure S5). Remarkably, a model comprising a “purely anisotropic interface” (i.e. a seismic discontinuity that cuts across a single rock formation) at about 3.9 km depth in the center of the dome fits the data, decoupling the signals on the k = 0 and k = 1 harmonics. Here, to model the observed features, an anisotropy magnitude higher than 30% is required, decreasing to about 20% toward the border of the dome (Fig. 2b and Supplementary Table S1).

The wide n-pulses near the dome center (20–30 km along the profile) can be modeled with extremely low *V*_*S*_, attributable to melted dome-like lens in the middle crust. The deeper crustal and upper mantle structure confirms a regional shallow Moho at about 20 km depth^30^ with a marked up-warp to 15–18 km depth in the dome center. The upper mantle rocks broadly displays a relatively low *V*_*S*_ values (*V*_*S*_ about 3.8–3.9 km/s) with respect to an anhydrous olivine composition, supporting the hypothesis of a pervasively hydrated upper mantle beneath Tuscany^28^.

## Discussion

The reconstruction of the main seismic discontinuities and elastic properties yield a simplified model of the Larderello geothermal field (Fig. 3d). In the center of the dome, the “k-horizon” strongly correlates with the bottom of the anisotropic materials (Fig. 3b) and is associated to purely anisotropic interface within the Pliocene granites, not related to any local lithological discontinuity^16,31^. In the peripheral region, the k-horizon seems to cross the seismic anisotropic volume and the interpretation is more problematic for two reasons. First, we notice that signal related to the k-horizon is less robust in such area. Moreover, steeply dipping faults, generating signals visible on the k = 1 harmonics, could mask the real depth-extent of the anisotropic volumes as mapped by converted phases^14,16^. We therefore focus our discussion on the center of the Larderello dome.

We infer that the anisotropic transition at the k-horizon marks the lower boundary of the vapor-bearing fractured rock matrix. Laboratory measurements indicate that the strong anisotropy (>20%) of rock found in field measurements requires the presence of open microcracks^32^. In fact, anisotropy could be related to the presence of biotite bearing rock (e.g. micaschists) widely spread throughout the area^18^, given by seismic wave propagation normal to the foliation plane^33^. Nervertheless, we can rule out this hypothesis as compilations of P-wave anisotropy values for such lithologies have not reported values larger than 22–25%^34^. P-wave anisotropy as high as 30% can uniquely be generated from vapor-bearing cracks, where liquid-filled cracks could explain maximum anisotropy of about 12% of S-wave anisotropy^19^. Remarkably, RFs have been proven to be more sensitive to P- than to S-wave anisotropy^25^.

Below the k-horizon, fluids traverse the supercritical conditions, as suggested from temperatures close to 350–400 °C frequently found at shallow depths in the area^18^, while, at the same depth level, diffuse microseismicity indicates still persistent, high rock permeability due to a network of open fractures (Fig. 3b). The abrupt termination at depth of the ultra-high anisotropic behaviour of the granites is associated with the different compressibility of the fluid filling the microcracks, supporting an increase of fluid bulk modulus *κ*_*f*_ of about two order of magnitude, from about 0.1–1 to >10 kbar^19^ as expected for H_2_ O traversing the supercritical point^6^ (see Supplementary Figure S6). The existence of the physical conditions for hosting SCF in Larderello area have been suggested from bottom-hole measurements (T = 300–360 °C and P = 20.0–25.0 MPa)^15,16^. Phase diagrams P-T for this geothermal field show that the concentration of NaCl plays a crucial role keep the saline brine to sub-critical state. Our results indicate that such concentration should be lower than 25%^17^.

The final transition from high- to low-permeability rocks occurs about 2 km depth below the k-horizon, coincident with the seismicity cut off (Fig. 3b). This depth is comparable with the depth estimated from laboratory measurements and, thus, the seismicity cut off can be explained as the transition to full plastic behavior of the granites^7^. The lateral extension of the volume where SCF have been found (about 10–12 km wide) is deeply linked to the location of the Quaternary igneous intrusion and to the geometry of the crust-mantle discontinuity, as indicated by similar widths for the ultra-low S-wave velocity zone in the centre of the dome and for the local up-warp of the Mohorovic discontinuity. Decoupling at depth of the quaternary igneous intrusion from the k-horizon has been also suggested from local earthquake tomography^31^ and measurement of electrical conductivity^16^.

We document abrupt changes in anisotropic properties within Pliocene granites generated by the occurrence of SCF. Seismic anisotropy within geothermal fields has been observed using S-wave splitting measurements^35^ Measurements of S-wave anisotropy as high as 10% have been widely interpreted as a marker for the presence of liquid-filled microcracks^36^, but studies based on S-wave splitting observations have less resolution on P-wave anisotropy, related to vapor-filled cracks. P-wave anisotropy have been observed using wide angle active seismic data^37^ and measured in samples collected in geothermal fields (up to 20% in Larderello)^38^. Until now, mapping the presence of vapor-filled microcracks from observations of P-wave velocity anisotropy at depth implies extensive and cost-expensive active seismic survey, but resolution given by active seismic techniques is generally low beneath high-reflectivity horizons^39^, compromising the ability of mapping the change from ultra-high anisotropic to isotropic materials.

We mapped the presence of ultra-high P-wave anisotropy analyzing data from a low-cost (<100 K Euro) passive seismic experiment, in an area where highly defined resolution of the seismic structure is limited to the uppermost 4–5 km depth due to the presence of shallow high-reflectivity horizons^16^. However, passive seismic data have some limitations given by their low-frequency content with respect to active seismic data. In fact, uncertainty on the depth of the mapped interfaces has been valuated to be not smaller than 2–300 m for RF^40^. Moreover, the thickness of the SCF-bearing horizons needs to be comparable to the characterist wavelenght of the passive seismic data (again, in the order of 0.5 km). Nevertheless, we proved that passive seismic data contain valuable information about the seismic properties of the buried anisotropic materials, and their location at depth, within geothermal fields.

Our results indicate that passive seismics help in resolving the transition from ultra-high anisotropic to isotropic materials at local-to-regional scale, defining the target depth of supercritical reservoirs. Thus, our discovery has important implications for the future of geothermal exploration aimed to locate supercritical resources. Although its vertical resolution hardly reaches that possible by active seismics, its low-cost and low-impact make passive seismics at the fore-front of the next generation of geophysical tools for investigating the shallow-crust^41^.

## Methods

### Receiver function

The analysis of teleseismic P-to-s converted waves (so called Receiver Function) is a long-established passive seismic tool for imaging Earth’s seismic structure from the upper-mantle^42^ to the shallow-crust (e.g. sedimentary basin)^43^. Radial and Transverse RF are time-series of converted waves, extracted from the P-wave coda through the deconvolution of the Vertical component from the Radial and Transverse components^20^. Transverse RF contains energy converted out of the radial plane, and is generally considered a proxy for anisotropy and/or dipping structures^44^. The analysis of the time-delay and amplitude of such converted waves can put constraints on the depth of the velocity contrasts where the P-to-s conversions occurred. In this study, we computed RF using a frequency-domain deconvolution^45^. Two different frequency bands have been analysed, 0.5 Hz and 1 Hz (see Fig. 2), limiting the vertical resolution to approximately 2–4 and 1 km, respectively. After visual inspection, we selected 1886 high S/N ratio RF, with minimum of 36 and maximum of 165 RF per station. In Supplementary Figure S1, we illustrated the data-set for a single station in the center of the Larderello dome, LA05. RFs have been stacked according to their back-azimuth (baz) and epicentral distance (dist), in bins of 20° baz and x 40° dist with a 50% overlapping scheme, to suppress noise and highlight continuity of P-to-s conversions. In Supplementary Figure S1a, we show the Radial and Transverse component of the RF data-set as a function of the back-azimuth of the incoming P-wave. Positive (negative) arrivals are marked in blue (red). Light green areas define standard deviation of the RF bins computed from stacking process. The presence of relevant energy on both components indicates the presence of highly anisotropic material or 3D structures at depth.

### Common-Conversion Point

To image the crustal structure at regional-scale length over the entire Larderello geothermal field, we implemented a Common Conversion Point algorithm (CCP)^46^. We followed the approach developed in^23^. We defines a NW-SE profile that runs parallel to the main active seismics lines used explore the deep crust in the area (CROP18A and B, see Fig. 1). The profile has been sampled at evenly spaced points (so called “spots”) every 2.5 km. For each spot, we define an rectangular area of 5 km along and about 15 km across the profile. RFs computed at single stations are stacked together if the surface projection of their piercing points at 15 km depth fall inside the rectangular area. To avoid blurring from incoherent stations given by the strict 3D geometry of the Larderello dome (almost cylindrical-symmetry), we only included data from the stations closest to the profile when creating the CCP images. In Supplementary Figure S2a, we present the results obtained for the spot along the profile at X = 35 km, both in terms of Radial and Transverse RF data-set, as a function of the back-azimuth of the incoming P-wave. Positive (negative) arrivals are marked in blue (red). Light green areas define standard deviation of the RF bins computed from stacking process. We observe the close resemblance to Supplementary Figure S1a obtained for a single station in the same area of the Larderello geothermal field.

### Harmonic decomposition

Both Radial and Transverse RF data-sets contain information about the elastic structure of the crust. Horizontal interfaces separating layers with different bulk seismic velocities generate P-to-s converted waves, recorded on the Radial RF data-set, which display no dependence from the back-azimuth (baz) of the incoming P-waves. Anisotropic layers at depth are responsible for P-to-s converted waves, recorded on both components, Radial and Transverse, which show strong dependence on the baz of the incoming P-waves. To separate the P-to-s converted energy generated by discontinuities in the bulk seismic velocity (”isotropic structure” hereinafter) from energy related anisotropic materials at depth (“anisotropic structure” hereinafter), we decompose our CCP images in angular harmonics^21,47^. The first harmonics, k = 0, contains the energy which displays no dependence on the baz of the incoming P-wave and is related to the “isotropic structure”, while the second harmonics, k = 1, contains energy which displays a 360° periodicity in baz. It is worth noticing that energy on the k = 1 harmonics could be related dipping interfaces as well. But dipping seismic discontinuities would generate periodic arrivals as a function of baz, on both the k = 0 and the k = 1 harmonics. Isolated arrivals on the k = 1 harmonics, as found in this study, can be uniquely associated to anisotropic materials at depth. Details on the harmonics decomposition can be found in^23^. In Supplementary Figures S1 b,c and S2b,c, we illustrate the harmonic decomposition of a RF data-set, for one single station and one spot along the profile, in the central region of the Larderello dome. The two data-sets contain similar, relevant arrivals on the k = 1 harmonics, pointing out the presence of anisotropic materials at depth at local- and regional-scale. Moreover, the strong similarity of the two k = 1 harmonics indicates that the CCP image is able to reproduce the broad features of the converted seismic wavefield. Errors on the k = 0 harmonics (light green shades in Fig. 2 and Supplementary Figures S1 b and S2b), have been computed adopting a bootstrap procedure as described in^48^. Here, we resampled the original data-set 100 times, and we computed the standard deviation of the resampled population for each time-step of the k = 0 harmonics.

### Monte Carlo inversion

We performed a Monte Carlo inversion for a quantitative estimation of the most probable Earth’ model for the shallow crust beneath the Larderello geothermal field. The inversion methodology, a Neighbourhood Algorithm (NA), has been described in^49^ and^27^. NA simultaneously samples a large number of promising regions of the parameter space to avoid being trapped in local minima. We adopted the same strategy presented in^21^. We ran an inversion for each spot along the profile and we presented the results in term of best-fit model found. For each spot, we sampled 21000 Earth’s model over 201 iterations. After a first iteration where 1000 samples are randomly drawn from the model space, we set the algorithm to sample 100 new models from the 10 best-fit Voronoi cells (see^49^ for details). Model parameterization has been defined according to the observations on the k = 0 and k = 1 harmonics from Fig. 2. In Supplementary Table S1, we reported the best-fit model plotted in Fig. 3 and used to compute the synthetics shown in Supplementary Figure S5.

### Local seismicity

Local earthquake locations are obtained using a non-linear, probabilistic procedure^50^ using travel-times calculated for the 3D tomographic model obtained by^26^. The catalog amounts to more than 2800 hypocentral solutions spanning the May 14, 2012 – August 31, 2013 time interval.

### Data availability

Continuous seismic recordings from permanent seismic stations are available on EIDA (http://www.orfeus-eu.org/data/eida/index.html). The seismic data recorded in the framework of RETREAT project (NSF grant EAR-0208652) can be freely download from IRIS data archive: http://ds.iris.edu/ds/nodes/dmc/data/. Part of the seismic data used for this work have been collected in the frame of an internal INGV project. and are embargoed till 31.12.2017. Afterward, continuous seismic data will be made available upon request to D. P. (davide.piccinini@ingv.it).

## Electronic supplementary material

Supporting data analysis

Interpolated k=0 harmonics

Interpolated k=1 harmonics
