# Supplementary material for: Discovering geothermal supercritical fluids: a new frontier for seismic exploration
Source: Sci Rep. 2017 Nov 6;7:14592. doi: 10.1038/s41598-017-15118-w (PMC5674042; doi:10.1038/s41598-017-15118-w)
Supplement: Supplementary file 1 — Supporting data analysis [file 41598_2017_15118_MOESM1_ESM.pdf]

SUPPLEMENTARY INFORMATION

TITLE: Discovering geothermal supercritical fluids: a new frontier for seismic exploration

Authors: Nicola Piana Agostinetti[1,2,*], Andrea Licciardi[3], Davide Piccinini[4], Francesco Mazzarini[4], Giovanni Musumeci[5], Gilberto Saccorotti[4], Claudio Chiarabba[6]

[1] Department of Geodynamics and Sedimentology, University of Vienna, Vienna, Austria

[2] Geophysics Section, Dublin Institute for Advanced Studies, Dublin, Ireland

[3] Géosciences Rennes, Univerisity of Rennes 1, Rennes, France

[4]Istituto Nazionale di Geofisica e Vulcanologia, Sezione di Pisa, Pisa, Italy

[5]Dipartimento di Scienze della Terra, Universita' di Pisa, Pisa, Italy

[6]Istituto Nazionale di Geofisica e Vulcanologia, Centro Nazionale Terremoti, Rome, Italy

[*] Corresponding author

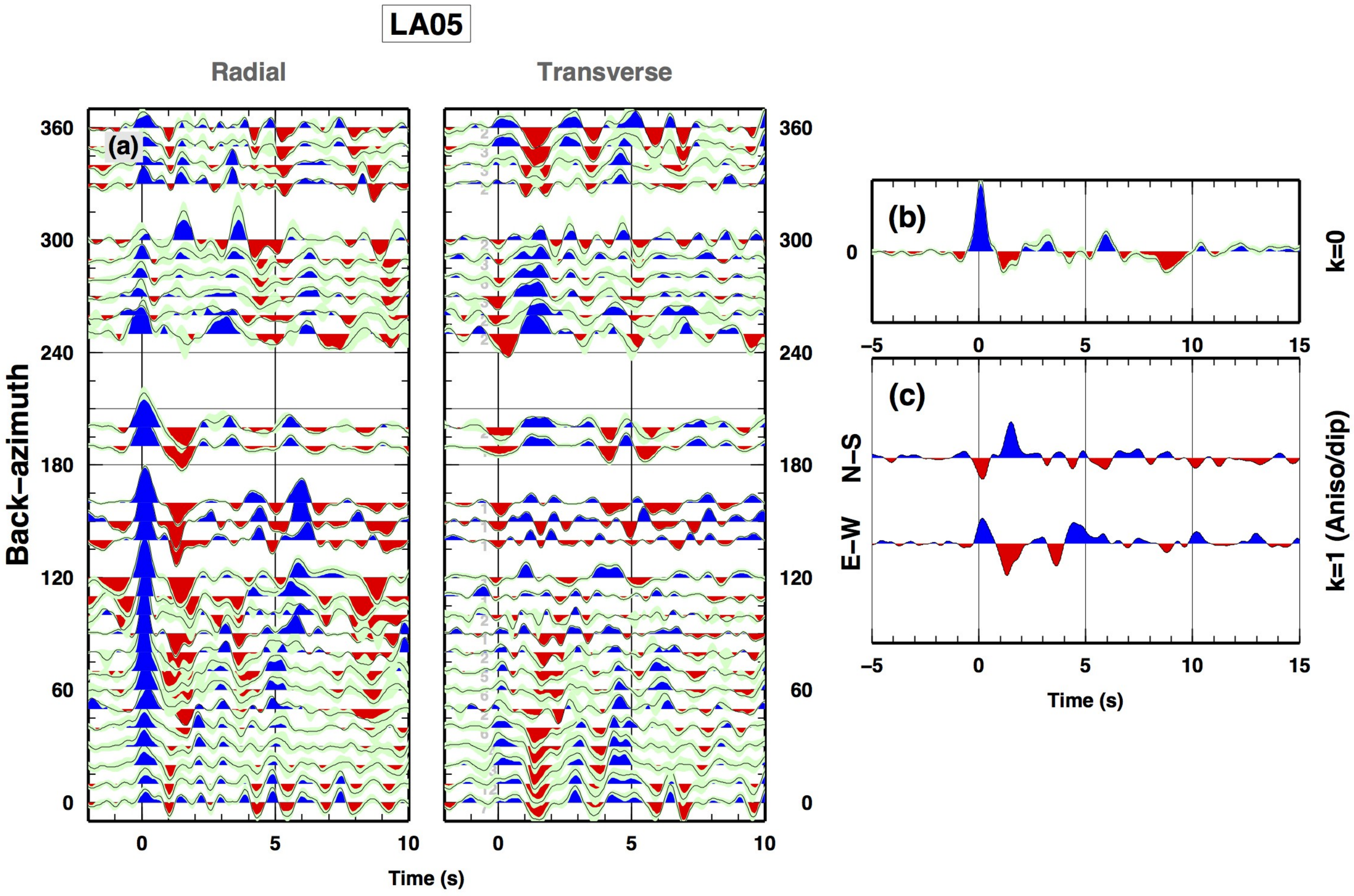

Supplementary Figure S1. RF data-set for one single station: LA05. (a) RF bins as a function of back-azimuth of the incoming P-wave. Positive (negative) arrivals are reported as blue (red) wiggles. Light green indicate standard deviation of the RF bins computed during stacking process. Left – Radial RF dataset. Right – Transverse RF dataset. (b-c) The k=0 and k=1 angular harmonics. Blue (red) colors as in panel (a).

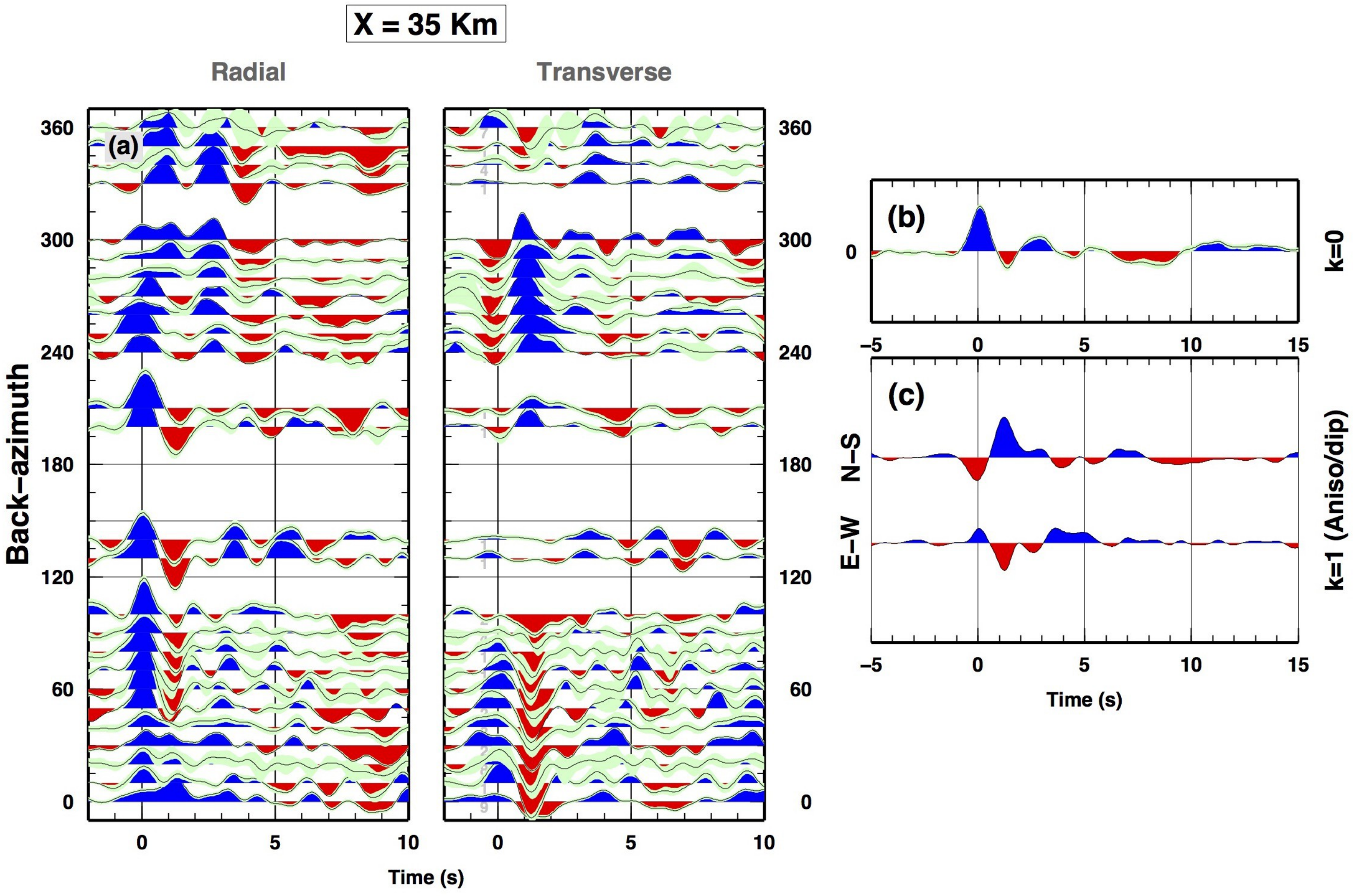

Supplementary Figure S2. Same as in Supplementary Figure S1, but for one point at X=35 km along the prof le.

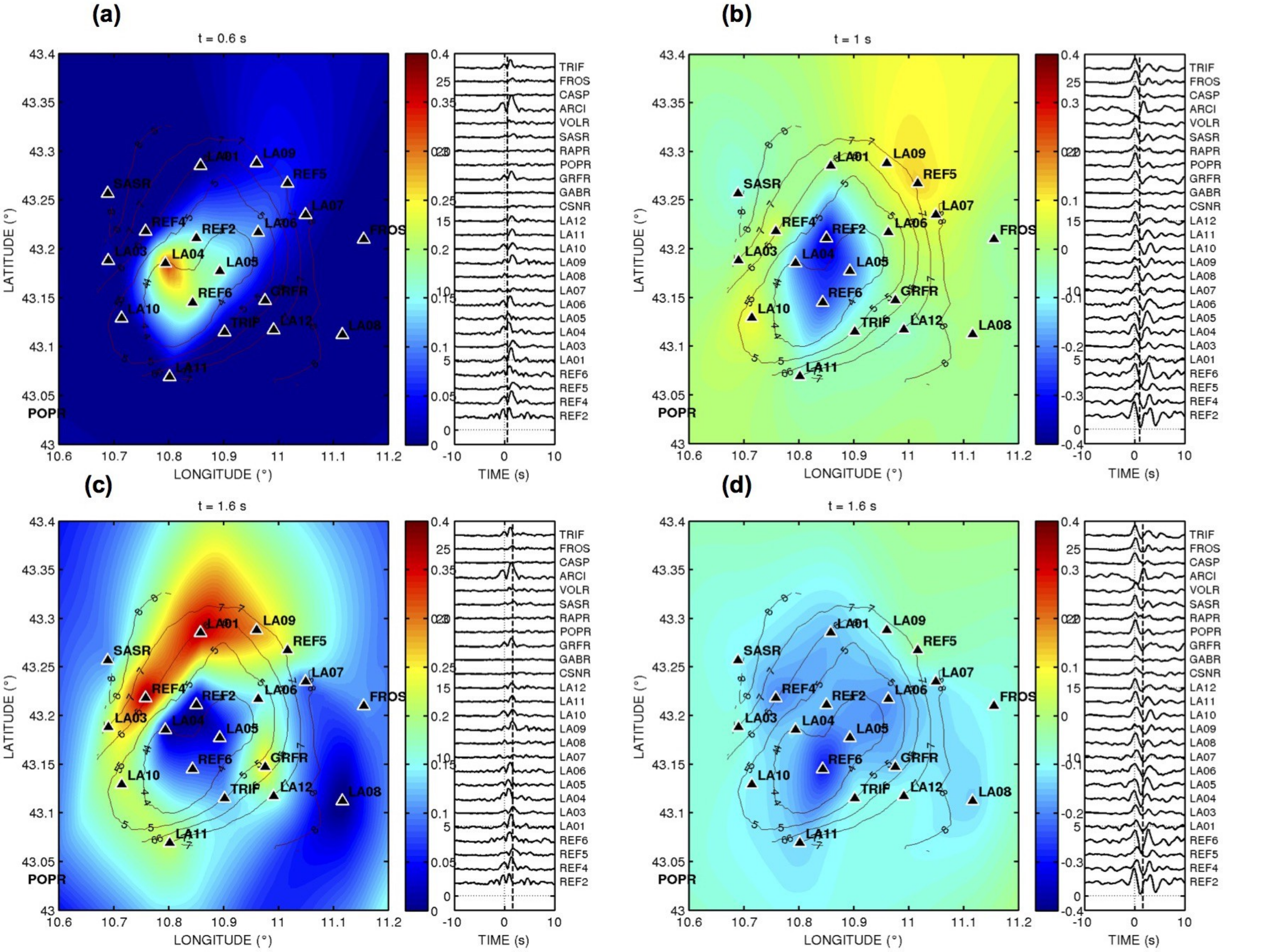

Supplementary Figure S3. Maps of the interpolated k=0 harmonics and energy on the k=1 harmonics at selected time-delays from direct P-arrival. In each panel, the colors indicate the interpolated values of the function from the results obtained for each single station (triangles). Iso-depth contour of the k-horizon as shown in Figure 1 are also reported. The k=0 harmonics and energy on the k=1 harmonics, for each station, are shown on the right of each panel, where a vertical dashed line indicates the selected time-delay. Map of the energy on the k=1 harmonics at 0.6 s (a) and at 1.6 s (c). Map of the k=0 harmonics at 1.0 s (b) and at 1.6 s (d).

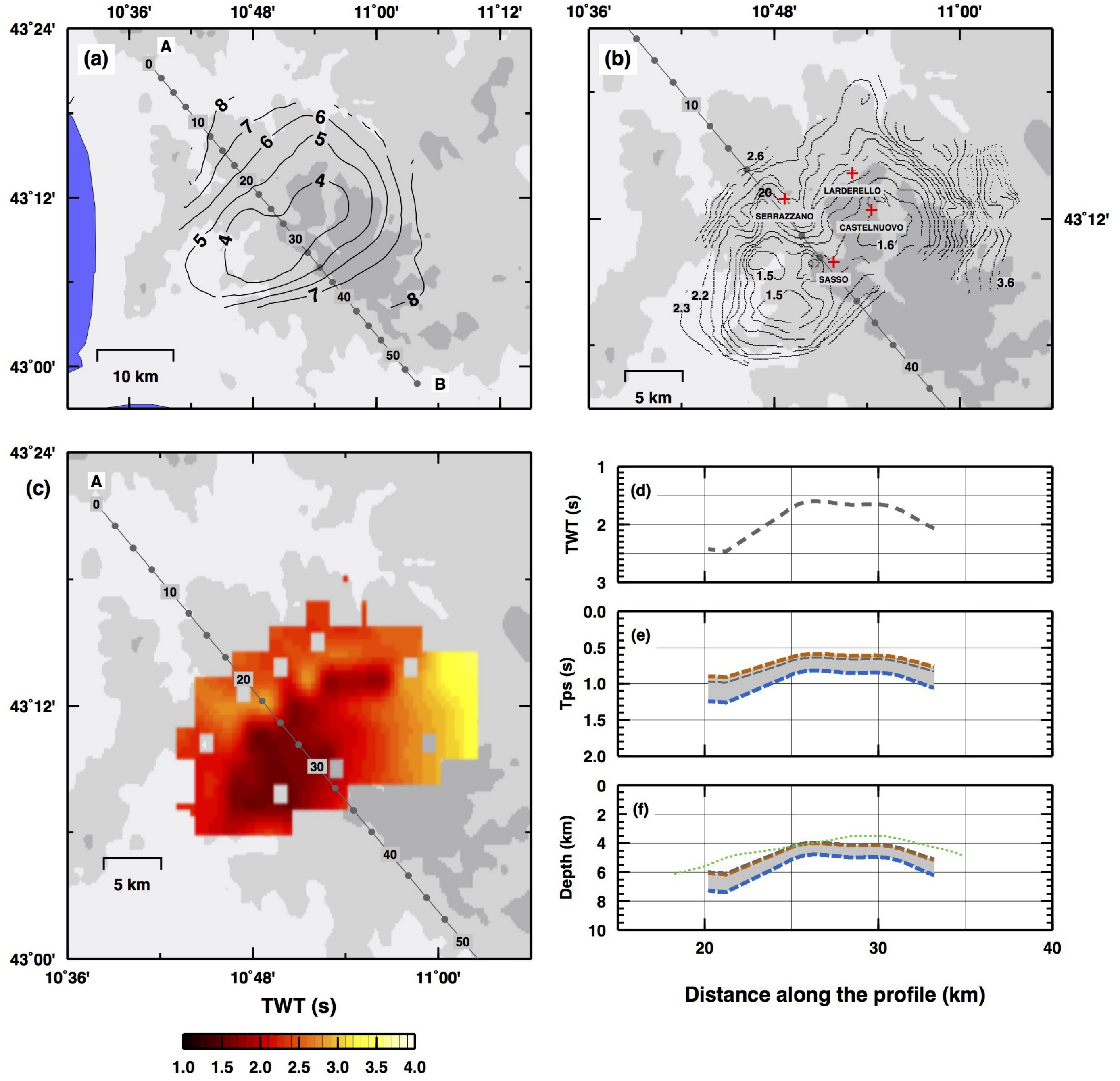

Supplementary Figure S4. The k-horizon along the prof le AB in Figure 1. (a) Low-resolution interpolated depth of the k-horizon of the entire study area[13]. (b) High resolution iso-contours of the two-way time (TWT) of the k-horizon in the center of the Larderello area from 396 data points[29]. Interpolated map of the TWT of the k-horizon from the data in panel (b). (d) Interpolated values of the TWT of the k-horizon along the prof le AB. (e) Estimated Tps for the k-horizon. Tps are computed from the interpolated values in panel (d), the formula in [43] and a simplif ied elastic model. We used two end-member models and the seismic model from the Monte Carlo inversion (Figure 3c). (e) Estimated depth of the k-horizon using two end-member models and the seismic model from the Monte Carlo inversion (Figure 3c). In panels (d) and (e), red dashed lines indicate the value computed using average shallow-crust model with Vp=5.0 km/s and Vp/Vs=1.72 [44]; a blue dashed line represents heavily-fractured carbonate rocks with Vp=6.0 km/s and Vp/Vs=2.0 [45]; and a grey dashed line shows the values computed using the model in Figure 4c. Minimum and maximum intervals based on the two end-member members models are shown as grey areas in panels (d) and (e). In panel (e), a green dashed line represents the low-resolution interpolated depth of the k-horizon from panel (a). Low-resolution interpolated depth and the depth-intervals def ined using TWT data are in good agreement.

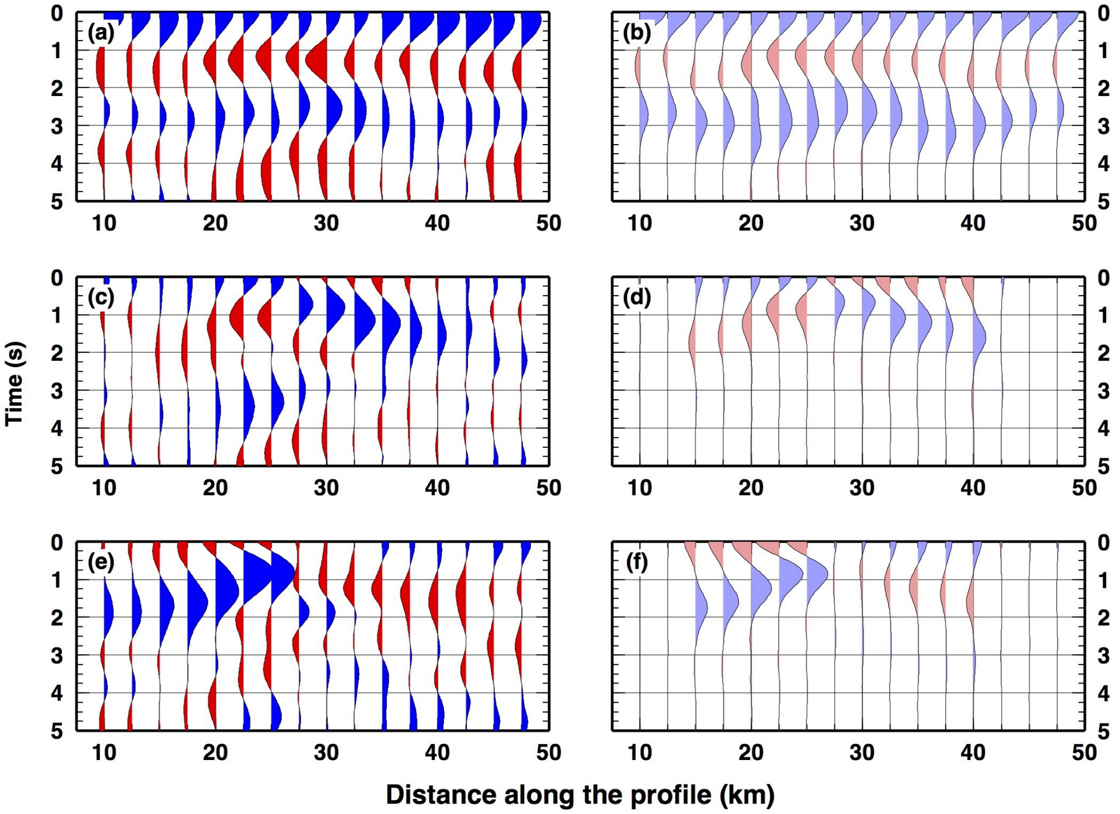

Supplementary Figure S5. Observed (left) vs synthetics (right) angular harmonics along the prof le AB. Synthetic harmonics are computed using the model in Figure 3c and Supplementary Table S1.

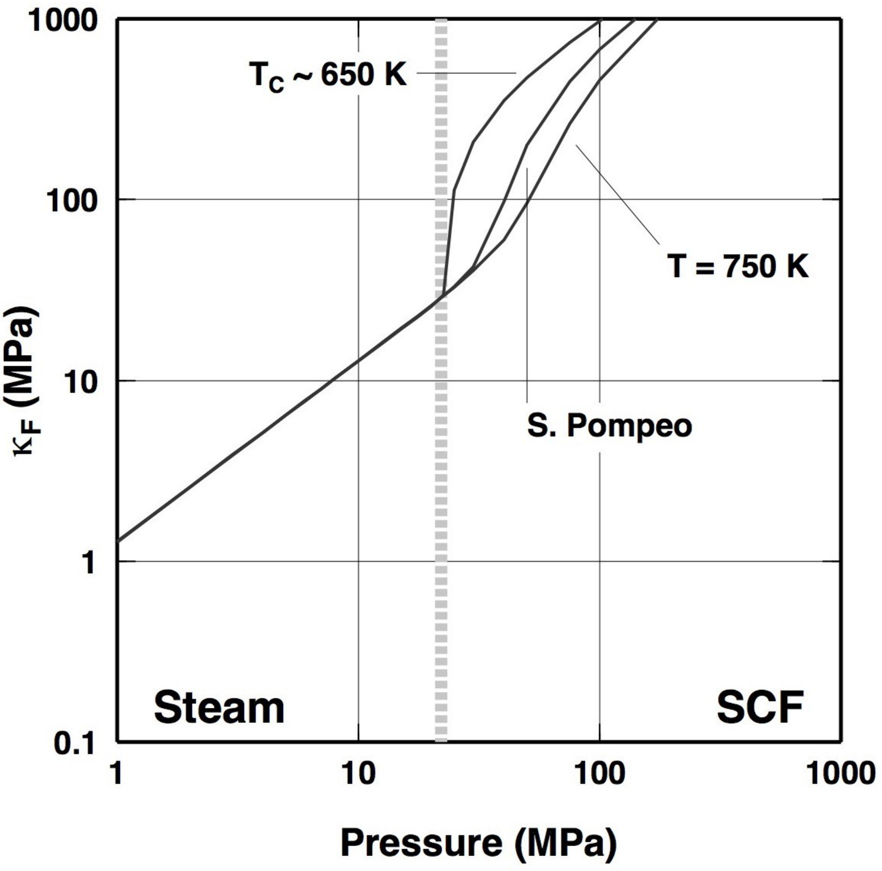

Supplementary Figure S6. Isothermal fluid bulk modulus k_f for pure water, as a function of confining pressure (three different temperature levels). Bulk modulus is computed from density and sound velocity values[20]. A vertical dashed grey line indicate super-critical pressure for H_2O. "Sam Pompeo" refers to the temperature found at the bottom of San Pompeo well (670+/25 K [15]).

Supplementary Movie M1. Movie of the k=0 harmonics interpolated over the entire area, from 0.1 to 2.0 s time-delay. In each frame, the colors indicate the interpolated values of the k=0 harmonics from the results obtained for each single station (triangles). Iso-depth contour of the k-horizon as shown in Figure 1 are also reported. The k=0 harmonics, for each station, are shown on the right of each panel, where a vertical dashed line indicates the selected time-delay. Time-frames at t=1.0 and t=1.6 s have been shown in Supplementary Figure S3**.**

Supplementary Movie M2. Movie of the energy of the k=1 harmonics interpolated over the entire area, from 0.1 to 2.0 s time-delay. In each frame, the colors indicate the interpolated values of the energy of the k=1 harmonics from the results obtained for each single station (triangles). Iso-depth contour of the k-horizon as shown in Figure 1 are also reported. The energy on the k=1 harmonics, for each station, are shown on the right of each panel, where a vertical dashed line indicates the selected time-delay. Time-frames at t=1.0 and t=1.6 s have been shown in Supplementary Figure S3.

Supplementary Table S1. Values of the parameters for the elastic models reported in Figure 3c. For each point along the profile, we present a stack of isotropic and anisotropic horizontal layers. Each layer is described in terms of: thick – thickness in kilometers; rho – density in kg/m3; Vs – S-wave velocity in m/s; Vp/Vs – ratio between P- and S- wave velocities; iso – a flag for identifying isotropic and anisotropic layers, which is equal to "1" for isotropic layers and "0" for anisotropic ones. %P and %S – percentage of anisotropy for P- and S- waves, respectively; trend – azimuthal direction of the symmetry axis for anisotropic layers; plunge – dip of the symmetry axis for anisotropic layers.

Additional References:

[53] Zhu, L., and H. Kanamori (2000), Moho depth variation in southern California from teleseismic receiver functions, J. Geophys. Res., 105(B2), 2969–2980, doi:10.1029/1999JB900322.

[54] Christensen, N.I. and Mooney, W.D., 1995,Seismic Velocity Structure and Composition of the Continental Crust: A Global View,J. Geophys. Res., 100, 9761-9788

[55] F. Trippetta, C. Collettini, S. Vinciguerra, P.G. Meredith (2010) Laboratory measurements of the physical properties of Triassic Evaporites from Central Italy and correlation with geophysical data, Tectonophysics, 492, 1, 121-132, http://dx.doi.org/10.1016/j.tecto.2010.06.001.

Table S1. Velocity models

```
#  -----------------------------------------------
#  Model for X Km along the profile                         X=10
#
#                                                   thick    rho      Vs   Vp/Vs  iso    %P   %S  trend  plunge
                                                     10.5   2600    3400    1.75    1     0    0      0       0
                                                      2.0   2600    3000    1.75    1     0    0      0       0
                                                      4.0   2600    2800    1.75    1     0    0      0       0
                                                      3.0   2600    3000    1.75    1     0    0      0       0
                                                      ----  3300    3850    1.81    1     0    0      0       0
#
#  -----------------------------------------------
#  Model for X Km along the profile                         X=12.5
#
#                                                   thick    rho      Vs   Vp/Vs  iso    %P   %S  trend  plunge
                                                      9.0   2600    3200    1.75    1     0    0      0       0
                                                      1.0   2600    2800    1.75    1     0    0      0       0
                                                      2.0   2600    2600    1.75    1     0    0      0       0
                                                      6.0   2600    2800    1.75    1     0    0      0       0
                                                      ----  3300    3850    1.81    1     0    0      0       0
#
#  -----------------------------------------------
#  Model for X Km along the profile                         X=15
#
#                                                   thick    rho      Vs   Vp/Vs  iso    %P   %S  trend  plunge
                                                      1.2   2600    2800    1.75    0    20   20    300      45
                                                      2.0   2600    2000    1.75    1     0    0      0       0
                                                      2.0   2600    2200    1.75    1     0    0      0       0
                                                      2.0   2600    2600    1.75    1     0    0      0       0
                                                      ----  3300    3850    1.95    1     0    0      0       0
#
#  -----------------------------------------------
#  Model for X Km along the profile                         X=17.5
#
#                                                   thick    rho      Vs   Vp/Vs  iso    %P   %S  trend  plunge
                                                     12.0   2600    2800    1.75    0    28   28    289      64
                                                      0.1   2600    2800    1.75    1     0    0      0       0
                                                      1.0   2600    2000    1.75    1     0    0      0       0
                                                      1.0   2600    1800    1.75    1     0    0      0       0
                                                      1.1   2600    2000    1.75    1     0    0      0       0
                                                      1.1   2600    2200    1.75    1     0    0      0       0
                                                      3.2   2600    2600    1.75    1     0    0      0       0
                                                      ----  3300    3850    1.75    1     0    0      0       0
#
#  -----------------------------------------------
#  Model for X Km along the profile                         X=20
#
#                                                   thick    rho      Vs   Vp/Vs  iso    %P   %S  trend  plunge
                                                      9.5   2600    2800    1.75    0    32   32    294      56
                                                      0.1   2600    2800    1.75    1     0    0      0       0
                                                      1.6   2600    1600    1.75    1     0    0      0       0
                                                      1.0   2600    1400    1.75    1     0    0      0       0
                                                      1.2   2600    1600    1.75    1     0    0      0       0
                                                      2.3   2600    2200    1.75    1     0    0      0       0
                                                      4.0   2600    2600    1.75    1     0    0      0       0
                                                      ----  3300    3850    1.75    1     0    0      0       0
#
#  -----------------------------------------------
#  Model for X Km along the profile                         X=22.5
#
```

Table S1. Velocity models

		thick	rho	Vs	Vp/Vs	iso	%P	%S	trend	plunge
#		thick	rho	Vs	Vp/Vs	iso	%P	%S	trend	plunge
		6.6	2600	2800	1.75	0	32	32	297	43
		1.5	2600	2800	1.75	1	0	0	0	0
		1.9	2600	1600	1.75	1	0	0	0	0
		1.0	2600	1400	1.75	1	0	0	0	0
		1.8	2600	1600	1.75	1	0	0	0	0
		1.1	2600	2200	1.75	1	0	0	0	0
		2.5	2600	2600	1.75	1	0	0	0	0
		----	3300	3850	1.75	1	0	0	0	0
#										
#	---									
#	Model for X Km along the profile				X=25					
#										
#		thick	rho	Vs	Vp/Vs	iso	%P	%S	trend	plunge
		6.3	2600	2800	1.75	0	32	32	298	50
		1.7	2600	2800	1.75	1	0	0	0	0
		1.1	2600	1600	1.75	1	0	0	0	0
		1.1	2600	1400	1.75	1	0	0	0	0
		1.8	2600	1600	1.75	1	0	0	0	0
		2.4	2600	2200	1.75	1	0	0	0	0
		2.3	2600	2600	1.75	1	0	0	0	0
		----	3300	3850	1.75	1	0	0	0	0
#										
#	---									
#	Model for X Km along the profile				X=27.5					
#										
#		thick	rho	Vs	Vp/Vs	iso	%P	%S	trend	plunge
		4.0	2600	2800	1.75	0	32	32	174	70
		4.2	2600	2800	1.75	1	0	0	0	0
		1.0	2600	1600	1.75	1	0	0	0	0
		1.0	2600	1400	1.75	1	0	0	0	0
		1.1	2600	1600	1.75	1	0	0	0	0
		1.2	2600	2200	1.75	1	0	0	0	0
		2.7	2600	2600	1.75	1	0	0	0	0
		----	3300	3850	1.75	1	0	0	0	0
#										
#	---									
#	Model for X Km along the profile				X=30					
#										
#		thick	rho	Vs	Vp/Vs	iso	%P	%S	trend	plunge
		4.4	2600	2800	1.75	0	32	32	168	66
		4.2	2600	2800	1.75	1	0	0	0	0
		1.1	2600	1600	1.75	1	0	0	0	0
		1.6	2600	1400	1.75	1	0	0	0	0
		1.0	2600	1600	1.75	1	0	0	0	0
		1.1	2600	2200	1.75	1	0	0	0	0
		2.0	2600	2600	1.75	1	0	0	0	0
		----	3300	3850	1.75	1	0	0	0	0
#										
#	---									
#	Model for X Km along the profile				X=32.5					
#										
#		thick	rho	Vs	Vp/Vs	iso	%P	%S	trend	plunge
		8.2	2600	2800	1.75	0	32	32	160	66
		8.0	2600	2800	1.75	1	0	0	0	0
		1.4	2600	2000	1.75	1	0	0	0	0
		2.0	2600	1800	1.75	1	0	0	0	0
		1.1	2600	2000	1.75	1	0	0	0	0
		1.0	2600	2200	1.75	1	0	0	0	0

```
                              2.7  2600   2600   1.75   1      0    0     0      0
                             ----  3300   3850   1.75   1      0    0     0      0
#
# -----------------------------------------------
# Model for X Km along the profile         X=35
#
#                           thick   rho     Vs  Vp/Vs  iso    %P   %S  trend  plunge
                              9.0  2600   2800   1.75   0     24   24   149     60
                              0.1  2600   2800   1.75   1      0    0     0      0
                              1.1  2600   2000   1.75   1      0    0     0      0
                              1.0  2600   1800   1.75   1      0    0     0      0
                              1.4  2600   2000   1.75   1      0    0     0      0
                              2.3  2600   2200   1.75   1      0    0     0      0
                              4.0  2600   2600   1.75   1      0    0     0      0
                             ----  3300   3850   1.75   1      0    0     0      0
#
# -----------------------------------------------
# Model for X Km along the profile         X=37.5
#
#                           thick   rho     Vs  Vp/Vs  iso    %P   %S  trend  plunge
                              9.5  2600   2800   1.75   0     12   12   142     48
                              0.1  2600   2800   1.75   1      0    0     0      0
                              1.1  2600   2000   1.75   1      0    0     0      0
                              1.0  2600   1800   1.75   1      0    0     0      0
                              1.1  2600   2000   1.75   1      0    0     0      0
                              2.5  2600   2200   1.75   1      0    0     0      0
                              4.0  2600   2600   1.75   1      0    0     0      0
                             ----  3300   3850   1.75   1      0    0     0      0
#
# -----------------------------------------------
# Model for X Km along the profile         X=40
#
#                           thick   rho     Vs  Vp/Vs  iso    %P   %S  trend  plunge
                              1.1  2600   2800   1.75   0     20   20   150     45
                              3.0  2600   2000   1.75   1      0    0     0      0
                              2.0  2600   2200   1.75   1      0    0     0      0
                              2.0  2600   2600   1.75   1      0    0     0      0
                             ----  3300   3850   1.95   1      0    0     0      0
#
# -----------------------------------------------
# Model for X Km along the profile         X=42.5
#
#                           thick   rho     Vs  Vp/Vs  iso    %P   %S  trend  plunge
                             11.9  2600   3200   1.75   1      0    0     0      0
                              1.0  2600   2800   1.75   1      0    0     0      0
                              2.9  2600   2600   1.75   1      0    0     0      0
                              3.8  2600   2800   1.75   1      0    0     0      0
                             ----  3300   3850   1.81   1      0    0     0      0
#
# -----------------------------------------------
# Model for X Km along the profile         X=45
#
#                           thick   rho     Vs  Vp/Vs  iso    %P   %S  trend  plunge
                              1.1  2600   3400   1.75   1      0    0     0      0
                              2.0  2600   3000   1.75   1      0    0     0      0
                              2.9  2600   2800   1.75   1      0    0     0      0
                              3.0  2600   3000   1.75   1      0    0     0      0
                             ----  3300   3850   1.81   1      0    0     0      0
#
```

Table S1. Velocity models

Model for X Km along the profile X=47.5
#

#	thick	rho	Vs	Vp/Vs	iso	%P	%S	trend	plunge
	10.7	2600	3400	1.75	1	0	0	0	0
	2.0	2600	3000	1.75	1	0	0	0	0
	3.2	2600	2800	1.75	1	0	0	0	0
	3.0	2600	3000	1.75	1	0	0	0	0
	----	3300	3850	1.81	1	0	0	0	0

#
