# Supplementary figures and images for: Discovering geothermal supercritical fluids: a new frontier for seismic exploration
Source: Sci Rep. 2017 Nov 6;7:14592. doi: 10.1038/s41598-017-15118-w (PMC5674042; doi:10.1038/s41598-017-15118-w)

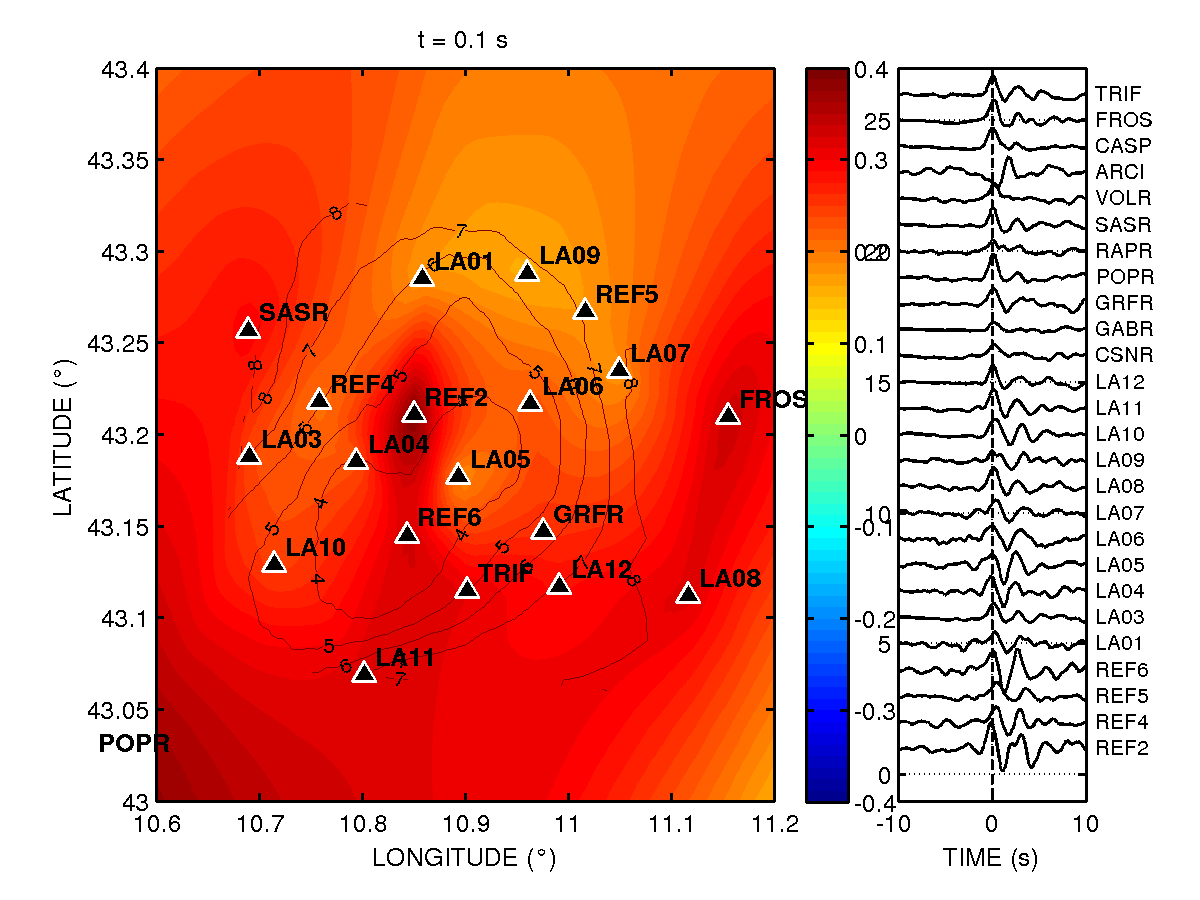

Supplement: Supplementary file 2 — Interpolated k=0 harmonics [file 41598_2017_15118_MOESM2_ESM.gif]

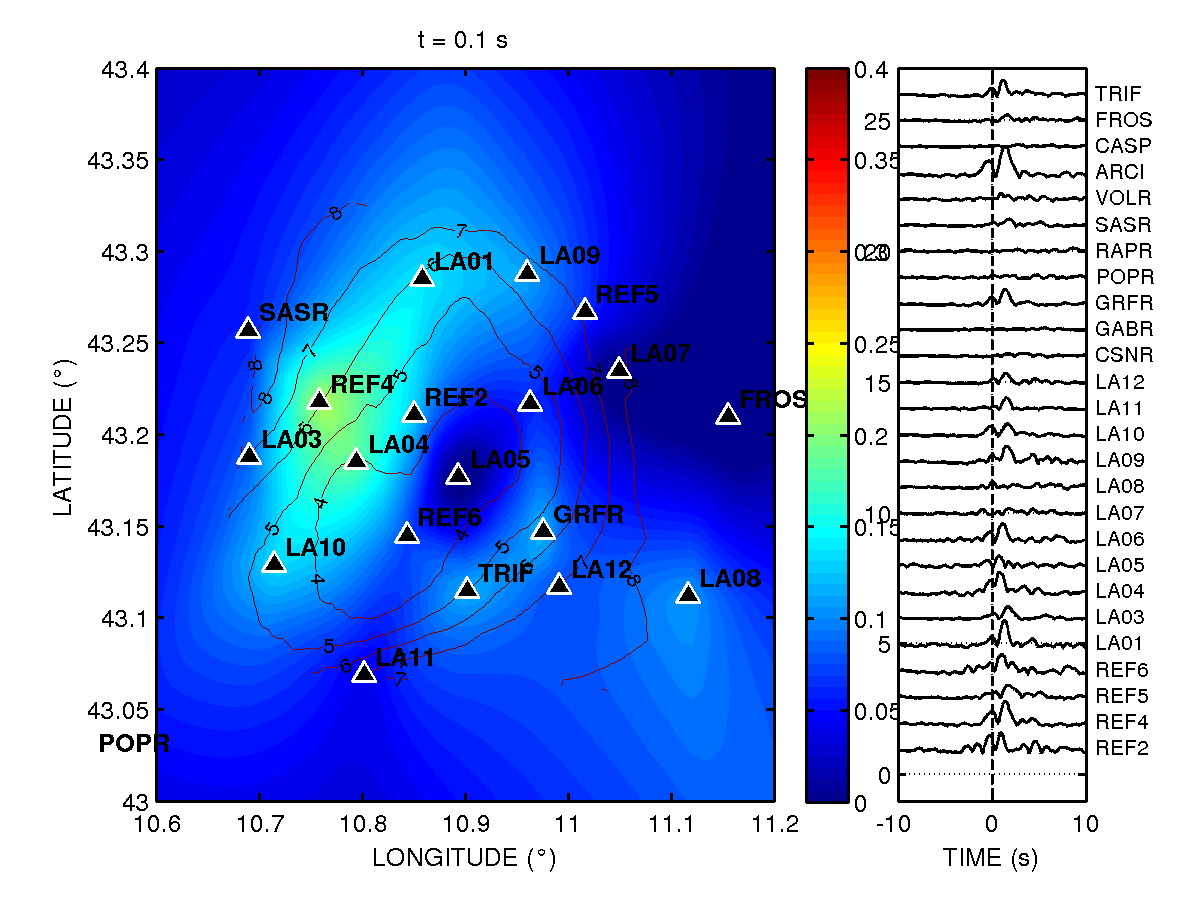

Supplement: Supplementary file 3 — Interpolated k=1 harmonics [file 41598_2017_15118_MOESM3_ESM.gif]
